# In vitro inhibition of SARS-CoV-2 Infection by dry algae powders

**DOI:** 10.1038/s41598-022-22148-6

**Published:** 2022-10-12

**Authors:** Daniel Garcia-Ruiz, Erendira Villalobos-Sánchez, David Alam-Escamilla, Darwin Elizondo-Quiroga

**Affiliations:** 1 Medical and Pharmaceutical Biotechnology Unit, Center for Research and Assistance in Technology and Design of the State of Jalisco (CIATEJ), Av. Normalistas 800, Colinas de la Normal, 44270 Guadalajara, Jalisco Mexico; 2 CREAMOS MAS S.A. DE C.V, Calle Monte Albán No. 965. Colonia. Independencia Oriente, C.P. 44340 Guadalajara, Jalisco Mexico

**Keywords:** Viral infection, Infection

## Abstract

*Chlorella* spp.,* Spirulina* spp., and fucoidan dry powders, are commercialized as food supplements and are considered safe for human consumption. Their broad-spectrum antiviral properties have been studied, however, their effect against SARS-CoV-2 remains unknown. We investigated the potential antiviral activity of three algae powders: *Chlorella vulgaris*, *Arthrospira maxima* (*Spirulina*) and fucoidan purified from marine brown algae *Sargassum* spp. against SARS-CoV-2 infection in vitro. Vero cells were incubated with 70 μg/ml of each algae powder and either 50 or 100 TCID_50_/ml of SARS-CoV-2, in two types of experiments (pretreatment and simultaneous) and comparing two kinds of solvents (DMEM and DMSO). *Chlorella vulgaris* powder, inhibited SARS-CoV-2 infection in all assays; viral RNA was significantly reduced in supernatants at 24, 48, 72, and 96 h post-infection, the highest difference in viral load (8000-fold) was observed after 96 h. *Arthrospira maxima* powder inhibited SARS-CoV-2 infection using 50 TCID_50_/ml for both experimental schemes, but protection percent was lower when viral inoculum was increase to 100 TCID_50_/ml; viral RNA decreased 48 h after infection, reaching a 250-fold difference at 72 h. Fucoidan powder partially inhibited SARS-CoV-2 infection since no CPE was observed in 62.5% of trated cultures in DMEM, but the antiviral activity was increased to 100% of protection when DMSO was used as solvent. All the algae samples showed high antiviral activity against SARS-CoV-2 with a SI above of 18. These results suggest that all three algae samples are potential therapeutic candidates for the treatment of COVID-19.

## Introduction

Coronavirus disease (COVID-19) is an infectious disease characterized by fever, sore throat, loss of smell, and in severe cases, breathing difficulty and chest pain^1^. In December 2019 a cluster of unknown-origin pneumonia cases were reported in the city of Wuhan, Hebei province^2^. The etiological agent was identified as a novel coronavirus soon after^3^. Since then, COVID-19 has spread worldwide and was declared a global pandemic on March 11, 2020 by the world health organization (WHO)^4^. At the time of writing, over 422 million cases and 5.8 million deaths have been reported worldwide^5^. COVID-19 is caused by the severe acute respiratory syndrome coronavirus-2 (SARS-CoV-2), a single stranded RNA virus belonging to the betacoronavirus genus^6^. SARS-CoV-2 has been shown to bind to several cell receptors including ACE2, CD147 and CD209. Viral entry is mediated by binding between the viral spike protein and the cell ACE2 receptor; binding triggers a conformational change in the spike protein structure ultimately leading to the fusion of the viral and cell membranes, thus promoting the internalization of the viral genome and associated proteins^7^. The genome of RNA viruses mutates quickly due to the action of the error-prone viral RNA polymerase. Fast mutation rates promote rapid viral diversification^8^. Rapid mutation in SARS-CoV-2, has led to the continuous emergence of new variants, prompting WHO to characterize them into variants of interest (VOI) and variants of concern (VOC), according to the perceived risk to public health. As of November 2021, WHO has declared 5 VOC: alpha, beta, gama, delta and omicron^9^. Mass immunization programs have been established worldwide with considerable success, especially in developed nations. To date 66% of the extended European area and 59.7% of the US population have received full immunization^10,11^. However, breakthrough infections in vaccinated individuals have been reported and correlated with both, the time from infection and infection with novel variants, thus, a future increase in breakthrough infection rates is likely^11–17^. The emerging variants and the increase of the breakthrough infections phenomenon has highlighted the need to develop novel antiviral compounds.

The human cell receptors DPP4, CD147, CD209L and ACE2 as well as cellular proteases such as trypsin and elastase have been proposed as candidates for the development of novel antivirals^7,18^. On the other hand several vascular plant compounds, including alkaloids, flavonoids, polyphenols, and tannins, have been reported to inhibit either SARS-CoV-2 replication, or the activity of viral functional components^19^. Epigallocatechin-3-gallate (EGCG), a flavonoid from tea, has been found to inhibit SARS-CoV-2 infection in vitro, possibly by inhibiting the activity of the virus 3CL-protease, responsible for the viral polyprotein maturation^20–22^. Naringerin, another flavonoid present in grapes, has been shown to inhibit SARS-CoV-2 infection in vitro^23^. Berbamine, an alkaloid involved in Ca2+ signaling, has also shown anti-SARS-CoV-2 activity in vitro^24^.

In some research, micro and macro algae have been shown to produce antiviral agents. For instance, Hayashi et al.^25^ found in *Spirulina platensis* (*Arthrospira platensis*) an antiviral compound (calcium spirulan) that inhibits the entry of the enveloped viruses; herpes simplex (HIV-1), human cytomegalovirus (CMV), measles virus, mumps virus, and influenza A virus. In 1998, Ayehunie et al. demonstrated that aqueous extract from *S. platensis* inhibited HIV-1 replication in human T-cell lines, peripheral blood mononuclear cells (PBMC), and Langerhans cells (LC)^26^. Later polysaccharide fractions of *S. platensis* were analyzed, showing strong antiviral activity against CMV, HSV-1, HSV-2, HSV-6, Pseudorabies virus (PRV), and human immunodeficiency virus type 1 (HIV-1)^27,28^. Jang and Park found that a compound isolated from *A. maxima*, inhibited HIV-1 infection in the human T cell line MT4^29^. A similar observation was found when testing a raw extract of *Chlorella peruviana*, which showed antiviral activity by inhibiting the replication of dengue virus serotype 2 (DENV-2), in Vero-76 cells^30^. In another study *C. vulgaris* polysaccharides presented antiviral activity against replication of grass carp reovirus (GCRV), in vitro and in vivo^31^. In terms of macroalgae, the species of *Sargassum henslowianum* and *S. naozhouense* have been reported to inhibit HSV-1 in vitro, by cytopathic effect (CPE) inhibition and reduction of plaque assay respectively^32,33^. According to the above, a possible antiviral treatment against SARS-CoV-2 could be obtained from some micro and macro algae^34^. Raposo et al. summarized the bioactivity and applications of sulphated polysaccharides from various algae, including antiviral activities against a variety of viruses^35^. Recently, Song et al. reported that the sulphated polysaccharides fucoidan and carrageenan, showed significant antiviral activities at concentrations of 3.90–500 μg/ml against SARS-CoV-2^36^. Even though the capacity to produce antiviral compounds and the antiviral activity of whole extracts is well established, to the best of our knowledge, there are no reports of their effect against SARS-CoV-2 for any of the algae studied herein. In this context, we decided to investigate the effect of whole dried *C. vulgaris* and *A. maxima*, and purified fucoidan Alquimar® obtained from Sargassum spp. against SARS-CoV-2 infection in vitro.

## Results

### Samples maximum non-cytotoxic concentration

To identify the maximum non-cytotoxic concentration (MNCC), three differents concentrations of each sample were tested (50, 70, and 100 μg/ml). The 50 and 70 μg/ml concentrations exhibited no detectable damage such as loss of confluence, cell rounding or vacuolization when compared to the untreated control. Some modifications in the cell monolayer were observed at 100 μg/ml, thus, the 70 μg/ml concentration was selected for further antiviral assays. MTT assays showed a cell viability of 81.8%, 75.3% and 79.7% for *C. vulgaris*, *A. maxima* and fucoidan respectively, 72 h post treatment at a sample concentration of 70 µg/ml.

### Antiviral assays

**Figure 1:**
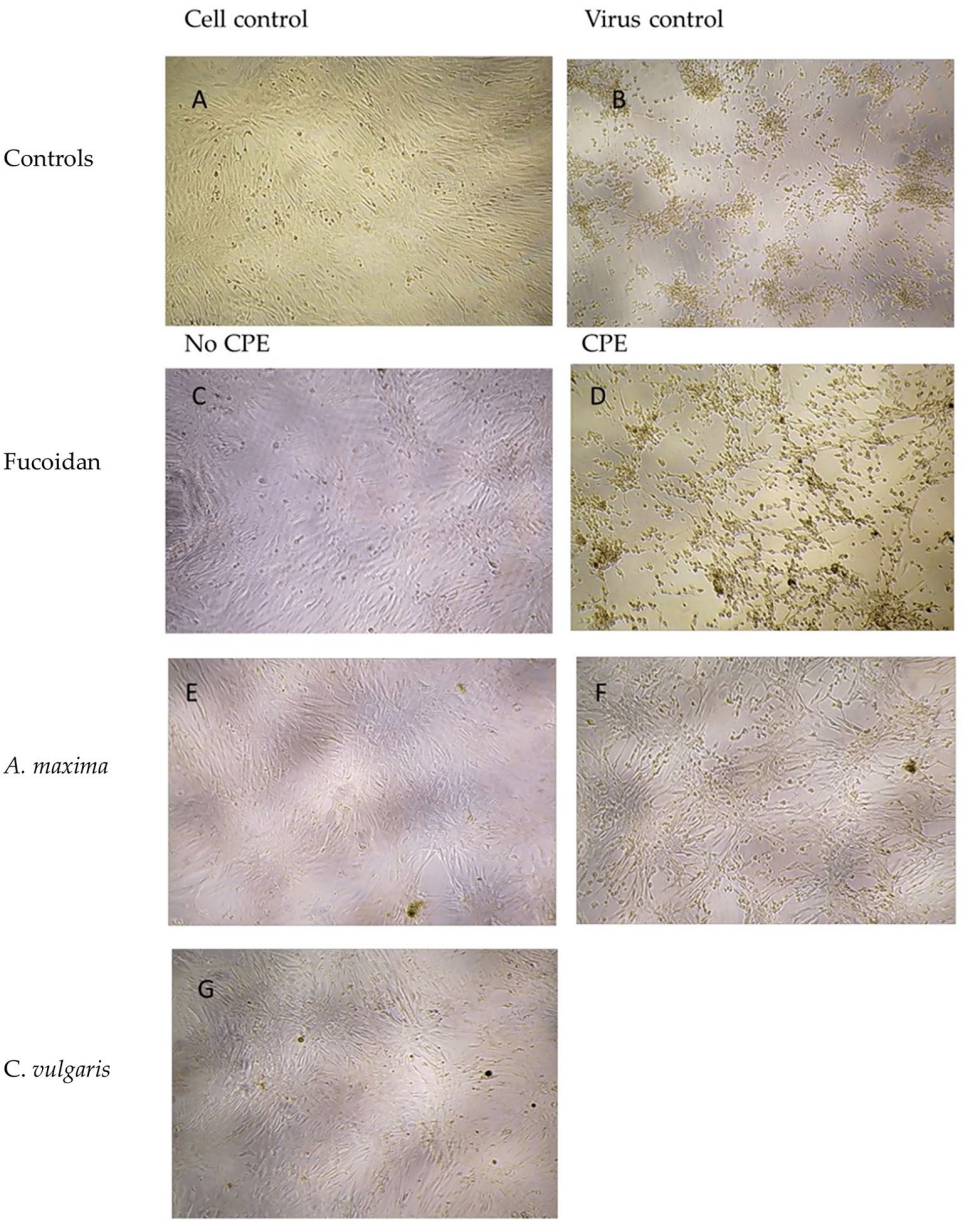
Inverted microscopic photographs representative of cultures at 96 h post infection. Vero cells were infected with 100 TCID_50_/ml of SARS-CoV-2 and treated with 70 μg/ml of algae. (**A**) Uninfected Vero cells; (**B**) Vero cells infected with SARS-CoV-2 presenting CPE consisting of cell detachment and culture degeneration; (**C**) Vero cells treated with fucoidan. Some of the cell cultures show CPE. (**D**) Cells treated with *A. maxima*, CPE is present in some of the cultures. (**G**) Cells treated with *C. vulgaris*, no CPE was detected in any of the cultures.

To determine the possible antiviral activity of the three different algae samples against SARS-CoV-2, two types of experiments were carried out (simultaneous and pretreatment), in presence of 50 or 100 TCID_50_/ml of SARS-CoV-2, plus powder samples dissolved in DMEM. In the assays in presence of *C. vulgaris* no CPE appearance was recorded for both types of experiments in any of the replicates, using 50 or 100 TCID_50_/ml of SARS-CoV-2 (Fig. 1). In the case of *A. maxima* at the same concentration, no CPE was observed when challenged with 50 TCID_50_/ml of the virus for both, simultaneous and pretreatment assays; however, when the viral concentration was increased to 100 TCID_50_/ml, CPE appeared in 50% and 75% of the replicates, for pretreatment and simultaneous assays respectively. Fucoidan assays at 70 μg/ml concentration also presented protection against viral infection; when challenged with 50 TCID_50_/ml, CPE appeared in 37.5% of the replicates for both pretreatment and simultaneous assays; however, when the viral concentration was increased to 100 TCID_50_/ml, CPE was observed in a 50% and 75% of the replicates, for pretreatment and simultaneous assays respectively (Table 1).

**Table 1:** Comparison in the percentage of infection (appearance of CPE) between 50 and 100 TCID_50_/ml.

Solvent	Sample	50 TCID_50_/ml		100 TCID_50_/ml	
		Pretreated (%)	Simultaneous (%)	Pretreated (%)	Simultaneous (%)
DMEM	*C. vulgaris*	0****	0****	0*	0*
	*A. maxima*	0****	0****	50	75
	Fucoidan	37.50*	37.50*	50	75
DMSO	*C. vulgaris*	0****	0****	0****	0****
	*A. maxima*	0****	0****	12.5**	0****
	Fucoidan	0****	0****	75	0****

All assays were carried out with 8 replicates, except for the DMEM assay with 100 TCID_50_/ml which had 4 replicates. The results are given in percentage of infection (CPE appearance in the replicates). 1-way-ANOVA ****p value < 0.00001. **< 0.001 *< 0.01.

### Algae antiviral activity depending on type of solvent

To assess if the solvent type could improve samples antiviral activity, experiments were carried out using DMSO as algae sample solvent, to contrast with results obtained using only DMEM. When *C. vulgaris* samples were challenged against 50 and 100 TCID_50_/ml, all samples showed 0% of infection, since no CPE appeared in any of the replicates, for both types of solvents, in pretreatment and simultaneous assays. In the case of *A. maxima*, in 50 TCID_50_/ml no CPE was observed for both types of solvents (D-MEM and DMSO) for pretreatment and simultaneous assays. When viral concentration was increased to 100 TCID_50_/ml using D-MEM as solvent, CPE appeared for both pretreatment and simultaneous assays (50 and 75% of infection respectively). However when *A. maxima* was diluted in DMSO, CPE only was observed in the pretreatment scheme (12.5% of infection) and no CPE appeared in simultaneous assay. In the case of fucoidan using 50 TCID_50_/ml of the virus when diluted in DMEM, CPE was observed in simultaneous and pretreatment assays (37.5% for both schemes), but in DMSO, no CPE was observed for both experimental schemes. However using 100 TCID_50_/ml of the virus and diluting the fucoidan in DMEM, CPE appeared in 50 and 75% for pretreatment and simultaneous assays respectively; but protection increased with DMSO as solvent, since no CPE appeared in simultaneous assay, but in pretreatment 75% of infection was observed (Table 1).

### Antiviral assays viral load

**Figure 2:**
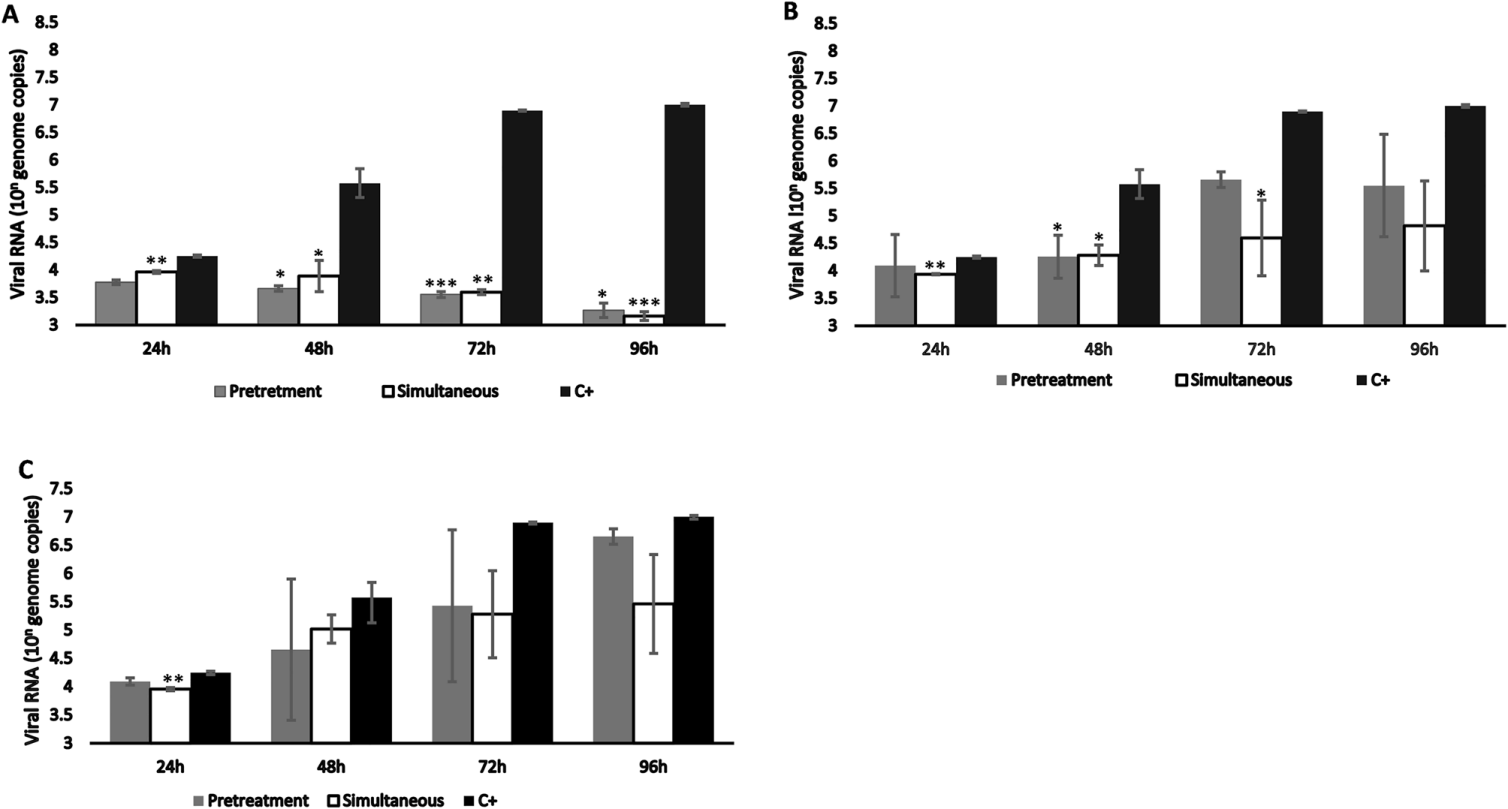
Quantification of viral load after infection. Mean values and mean ± SEM are shown. (**A**) *C. vulgaris*. (**B**) *A. maxima*. (**C**) fucoidan. Statistical significance is indicated by *P value < 0.01, **P value < 0.001 and ***P value of < 0.0001.

To further investigate the antiviral effect of the different algae samples on SARS-CoV-2 infection, viral genome copies were quantified by RT-qPCR. Three representative supernatants of each experiment showing presence or absence of CPE, were collected for the PCR assay. When infected with a MOI of 0.003, viral load increased in supernatants of untreated cells (positive control) reaching a plateau 72 hpi (Fig. 2A–C black bars). *C. vulgaris* strongly inhibited SARS-CoV-2 infection in vitro, in fact, a slight decrease in viral load was observed over time with the lowest concentration 10^3.2^ for pretreatment and 10^3.1^ genomic copies for simultaneous assays were measured at 96 hpi. The biggest inhibition was measured at 96 hpi with both pretreatment and simultaneous experiments showing 8000-fold decrease in viral load, this difference was statistically significant with Student’s t-test *p* values of < 0.0001 in the simultaneous assays and < 0.01 in the pretreatment assays (Fig. 2A).

RT-qPCR of supernatants recovered from cells infected with 100 TCID_50_/ml and treated with *A. maxima* powder diluted in DMEM in simultaneous assays, showed a sharp reduction in genome copy numbers with respect to the control. The highest difference was observed at 72 h, where an average of 10^4.5^ genome copies were estimated for cells treated with the algae, in contrast to an average of 10^6.9^ estimated for the positive control; this difference was statistically significant with a *P* value of < 0.01 (Fig. 2B). High variability between replicates was observed due to infection of some of the cultures.

Supernatants of cells treated with fucoidan in the simultaneous scheme showed a decrease in viral RNA 24, 48, 72 and 96 h post- infection (Fig. 2C), the strongest decrease (> tenfold) was measured at 72 h. Viral RNA also decreased in cell cultures in the pretreatment scheme at 48 and 72 h post-infection, however, these differences are not statistically significant.

### Algae dose–response effect

**Figure 3:**
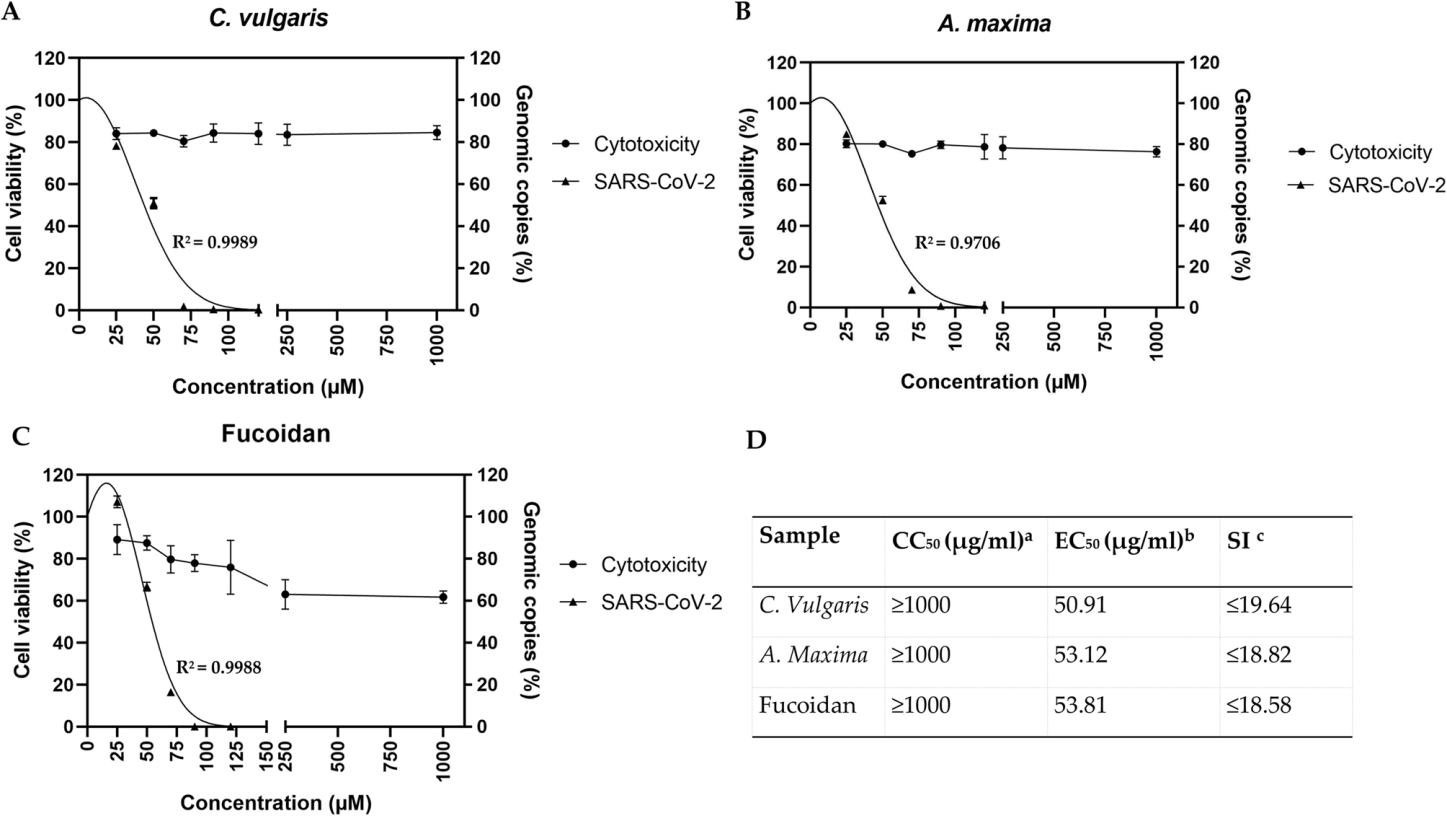
Algae doses-response activity against SARS-CoV-2. Vero cells were infected with 100 TCID_50_ of SARS-CoV-2. Data was confirmed experimentally in two independent experiments n = 4. Algae cytotoxicity measured as the percentage of cell viability at a given sample concentration was estimated by MTT. (**A**) *C. vulgaris*; (**B**) *A. maxima*; (**C**) Fucoidan; error bars show SD. (**D**) Selectivity index. a: 50% cytotoxic concentration. b: 50% efective concentration measured as differences in genomic copies between samples and positive control. c: Selectivity index: ratio between CC_50_ and EC_50_. Right Y axis shows genomic copies percent of SARS-CoV-2 infected experimets, compared to positive control. Left Y axis shows cell viability percent estimated for cytotoxicity experiments.

In order to find the selectivity index (SI) the 50% effective concentration (EC_50_) and the 50% cytotoxic concentration (CC_50_) were estimated. For the EC_50_ simultaneous antiviral assays were performed with increasing sample concentrations (1–120 µg/ml), against 100 TCID_50_/ml of SARS-CoV-2. The EC_50_ results were 50.91, 53.12 and 53.81 µg/ml for *C. vulgaris*, *A. maxima* and fucoidan respectively. To estimate the CC_50_ a 0–1000 µg/ml range of each sample was tested, using MTT assay. Results obtained by MTT showed that a concentration of 1000 µg/ml had a cellular viability of 86, 76, and 62% for *C. vulgaris*, *A. maxima* and fucoidan respectively. A range from 0 to 1000 µg/ml for all powder samples, does not produce cytotoxicity and is safe to use on cell cultures with a CC_50_ ≥ 1000 µg/ml (Fig. 3A–C). The SI was estimated as CC_50_/EC_50_ with results ≥ 19.64, ≥ 18.82 and ≥ 18.58 for *C. vulgaris*, *A. maxima* and fucoidan respectively (Fig. 3D).

## Discussion

In recent years, the use of dry Chlorella and Spirulina as food supplements and nutraceuticals has increased substantially, both Chlorella and Spirulina are considered an excellent source of non-animal protein and are used widely as a dietary supplement, especially in vegan diets^37^. These algae have not only been proved safe for human consumption but are also easy to produce and available at a low price worldwide. The results presented herein suggest that they also have potential to be used as virus inhibitors against SARS-CoV-2.

*Chlorella vulgaris* powder showed strong protection of the Vero cells cultures for all kinds of assays, solvent used, and viral concentration, since no CPE was observed in any replicates. Interestingly, the viral load measured in the supernatants of cell cultures showed sustained decrease from 10^3.7^ and 10^3.9^ at 24 hpi to 10^3.2^ and 10^3.1^ viral genome copies at 96 hpi for pretreatment and simultaneous experiments respectively. This decrease suggests that *C. vulgaris* inhibits SARS-CoV-2 replication since the estimated viral load consistently decreased over time. These results are in line with previous studies of animal viruses, such as grass carp reovirus and cyprinid herpesvirus 3^31,38^ where chlorella was shown to inhibit viral replication.

*Arthrospira maxima* powder also showed protection of cell cultures, no CPE was detected when 70 µg/ml of dry algae powder, dissolved in DMEM medium or DMSO, were added to cultures challenged with 50 TCID_50_/ml of SARS-CoV-2. However, it only showed partial protection when the viral concentration was increased to 100 TCID_50_/ml. The viral load present in supernatants of cells challenged with 100 TCID_50_/ml showed a great variability among replicates, this is probably due to viral infection of some replicas since *A. maxima* only provided partial protection at this viral concentration.

The dry fucoidan powder showed a minor antiviral activity compared to *C. vulgaris* and *A. maxima*. However, it is important to note that fucoidan did show antiviral activity against SARS-CoV-2. When fucoidan was dissolved in DMEM medium, infection was observed only in 37.5% cultures infected with 50 TCID_50_/ml. Infection rate decreased to 0% when DMSO was used as solvent. This suggests that fucoidan bioavailability might be better in organic solutions such as DMSO especially considering that fucoidan is a water-soluble polysaccharide. Our results are in line with those of Song et al. who previously reported fucoidan’s inhibitory effect against SARS-CoV-2^36^. Hidari found that algae derived polysaccharides inhibit DENV replication by preventing cell internalization^39^. In accordance with this, Elizondo-Gonzalez found that fucoidan blocked early stages of Newcastle Disease virus infection^40^. It is possible that fucoidan might also be preventing SARS-CoV-2 infection by preventing viral internalization. It is also worth noting that fucoidan has been found to promote recovery of mitochondrial membrane potential in PBMCs from COVID-19 recovered patients, indicating that fucoidan may be a potential treatment to diminish long-term sequelae of the disease^41^. This is particularly promising considering that fucoidan is isolated from algae that produce environmental disruption and pollution on Mexican coasts. With our results, we demonstrated that these algae improved their antiviral activity, when they were dissolved in DMSO, compared with the antiviral activity recorded when DMEM was used as solvent. Similar results were presented by Hernandez-Corona^28^, who inhibited 200 TCID_50_/ml of HSV-1 and HSV-2 using 69 and 300 µg/ml of *A. maxima* respectively.

According to results published by Ogando et al*.* SARS-CoV-2 replication kinetics show a logarithmic growth in virion progeny release, reaching a plateau 12 hpi when infected with a high viral concentration ( MOI of 3), assessed by plaque forming units^42^. In our experiments we used a 0.003 MOI (100 TCID_50/ml_) that produced a plateau at 72 hpi, assessed by RT-PCR. Other research groups have used similar viral loads as we used for our experiments to measure antiviral activity^28,33,36^. It would be interesting to performed further experiments using higher viral loads to understand if the algae tested herein, could have the antiviral activity in early stages of infection. Using a small viral load for antiviral assays, could result in a magnification effect after several infection cycles, due cumulative antiviral activity of each cycle; however, this seems to indicate that antiviral effect of the algae tested herein remains active for several replication cycles. Also a high viral concentration could conceal the antiviral effect of the compounds being tested, when CPE appearance is used to assess antiviral activity. Using a low viral concentration for infection, allows for a long replication time and thus a better opportunity to observe the viral CPE and for the antiviral compound action.

The three samples inhibited the infection of SARS-CoV-2 with a selectivity index above 18. According to Indrayanto et al*.*^43^, the ideal treatment should have a SI above 10. This finding is similar to the one reported by Hernandez et al*.*^28^ for *A. maxima* against Herpes Simplex Virus type 1 and 2 (HSV-1 and HSV-2) whose SI were 26 and 128 respectively. Magdalena et al*.*^44^ reported for *C. vulgaris* and *A. maxima* against Mayaro virus (MAYV) SI of 51.16 and 25.5 respectively^28,45^.

Taken together the data provided by this work, demonstrate that *C. vulgaris, A. maxima* and purified fucoidan Alquimar® obtained from *Sargassum* spp. powders, are potent inhibitors of SARS-CoV-2 in vitro. Further characterization is needed to identify the specific agents of each alga responsible for the antiviral activity and their action mechanism. Finally, the results of the present investigation suggest that these algae may have potential use in the treatment of SARS-CoV-2 infections; however, further research, preclinical and clinical studies are needed to support this assumption.

## Materials and methods

All experiments involving SARS-CoV-2 were carried out in the CIATEJ BSL-3 facility. The SARS-CoV-2 strain used in the experiments was a B.1.1 (pangolin nomenclature version 4) clinical isolate provided by the Hospital Civil de Guadalajara Fray Antonio Alcalde (Genbank access: ON457663.1), identified by RT-PCR using the primers and probes described by WHO for the diagnostic detection of the E and RdRp genes of the SARS-CoV-2 virus.

Vero CCL-81 cells (American Type Culture Collection) were maintained with Dulbecco’s Modified Eagle Medium (DMEM) containing l-glutamine (30 μg/ml, Sigma-Aldrich) and 10% of Fetal Bovine Serum (FBS) for growth medium (GM) or 2% of FBS for maintenance medium (MM) at 37 °C in a humidified atmosphere with 5% CO_2_.

To evaluate a possible antiviral effect against SARS-CoV-2, powder samples of *C. vulgaris, A. maxima* and a sample of fucoidan Alquimar ® (250.61 kDa) described by Díaz-Resendiz et al*.*^35^, all provided by the company Creamos mas, were used in this study. Each algae was rehydrated with 1 ml of DMEM or 1 ml of dimethyl sulfoxide (DMSO) to prepare a 10 mg/ml stock solutions. Samples were further diluted in different working concentrations using DMEM. In the case of experiments using DMSO as solvent, stock solution was diluted in DMEM to a final concentration of ≤ 4% DMSO.

To identify the MNCC for the experiments, three different concentrations were tested (50, 70, 100 µg/ml) in Vero cells. Cytotoxicity was determined microscopically by observation of cell morphological changes under inverted microscope. To confirm results of the selected concentration, cytotoxicity was measured by the MTT assay to determine the capability of living cells to convert a soluble tetrazolium salt[3-(4,5-dimethylthiazol-2-yl)-2,5-diphenyltetrazolium bromide (MTT) to insoluble formazan crystals. Vero cells (300,000 cells/ml) were seeded in 96-well plates 24 h before assay. Then the cells were treated with 100 μl of each sample at 70 μg/ml concentration in MM, later the plate was incubated for 48 h to determine the optimal effect of samples. Subsequently, the plate was carefully emptied, 10 μl/well of MTT solution (0.5 mg/ml) were added and the plate was incubated for 2 h. After incubation, 100 μl of isopropanol were added to each well and gently mixed until all crystals had dissolved. Once formazan was re-solubilized, absorbance was measured at 570 nm in a microplate reader to determine concentration. Control cells were incubated without samples. The percentage of cytotoxicity was calculated as (A − B)/A × 100, where A is the mean optical density of untreated wells and B is the optical density of wells with algae samples.

In order to assess the algae antiviral protection against SARS-CoV-2, two types of experiments were carried out using Vero cells. The first experiment was designated as “simultaneous” (70 μg/ml of sample, plus 50 or 100 TCID_50_/ml of the virus incubated 1 h at 37 °C, then added to Vero cells monolayers leaving the samples and the virus during 96 h at 37 °C and 5% of CO_2_ atmosphere.). The second experiment (designed to asses if previous cells exposure to the different samples could enhance antiviral activity) was designated as “pretreatment” (Vero cells monolayers were pretreated with 70 μg/ml of sample for 48 h, then the medium was discarded and 70 μg/ml of fresh sample, plus 50 or 100 TCID_50_/ml of the virus were added, leaving the samples and the virus during 96 h at 37 °C and 5% of CO_2_ atmosphere). Both the “simultaneous” and “pretreatment” experiments were performed using the rehydrated samples (either in DMEM or 4% DMSO plus DMEM) at 10 mg/ml concentration. All experiments were performed in 96-well plates kept in an incubator at 37 °C with 5% CO_2_ for 5 days and examined daily under the inverted microscope for evidence of viral CPE. Images were captured with a camera Optikam WiFi—4083.

To assess viral load and CPE inhibition between cell cultures treated with the algae samples, compared to untreated cultures (virus only), viral infection kinetics of simultaneous and pretreatment experiments were performed, using 100 TCID_50_/ml of the virus, plus 70 µg/ml of each sample. For this, cell culture supernatants were carefully harvested from each well of the 96-well microplates, at 24, 48, 72, and 96 h post-infection of each treatment, and kept in an ultra-freezer at − 80 °C for further use. For viral load quantification, the harvested supernatants from each treatment (by triplicate) per kinetic day, were thawed and RNA extraction was performed, using the QIAamp Viral RNA kit (QiagenTM, Hilden, Germany), according to the manufacturer instructions. The extraction was carried out from 100 µl of supernatant and the RNA was resuspended in 60 µl of Rnase-free water and stored at − 80 °C for later use.

Since greater differences in CPE appearance during the antiviral assays were recorded when 100 TCID_50_/ml were used for viral quantification, supernatants of the cultures from these experiments were collected to measure viral load. RT-qPCRs were performed using the primers and probes described by WHO for the diagnostic detection of the SARS-CoV-2 that amplify a 113 nt region of the virus E gene; (forward E_Sarbeco_F1 5′ ACAGGTACGTTAATAGTTAATAGCGT 3′, reverse E_Sarbeco_R2 5′ ATATTGCAGCAGTACGCACACA 3′ and prove E_Sarbeco_P1 5′ FAM-ACACTAGCCATCCTTACTGCGCTTCG-BHQ1 3′) approved for its use in Mexico by Instituto de Diagnóstico y Referencia Epidemiológicos Dr. Manuel Martínez Báez (InDRE). Super ScriptTM III PlatinumTM One-Step Qrt-PCR System kit (Invitrogen) was used to perform the RT-PCRs in a CFX96 Real-Time System thermocycler instrument (Bio-Rad). To quantify the treatment viral loads, a standard curve with four triplicated dilutions was generated, using a plasmid containing SARS-CoV-2 genome fragments, recognized by the envelope gene probe, provided by IBT, UNAM. Finally, to estimate the viral load, the average Ct of each dilution was used to perform a simple linear regression.

To understand the algae dose–response effect against the virus, the EC_50_ (the sample concentration in which genomic copies are reduced 50% compare to positive control), was estimated performing an antiviral activity assay under the simultaneous scheme, using 5 different algae concentrations diluted in DMEM plus 4% DMSO (25, 50, 70, and 120 µg/ml) against 100 TCID_50_ of SARS-CoV-2. Plate was incubated at 37 °C and 5% CO_2_, cells were monitored daily for presence of cytopathic effect.

To find the selectivity index (SI) a 0–1000 µg/ml range of each sample was tested, using MTT assay to asses sample 50% cytotoxic concentration (CC_50_; the concentration with a 50% reduction in cell viability) and the selectivity index was estimated as the relation between EC_50_ and CC_50_.

Data normality was assessed using Shapiro Wilk normality test. The statistical significance of control and treatment groups, were assessed using a one-way ANOVA for antiviral assays. Student’s t-test for PCR results were carried out using IBM SPSS statistics. A *p* value < 0.05 was considered statistically significant. GraphPad Prism V.8.02 was used to generate graphics and calculate the EC_50_ of the samples, using non-linear regression.

### Institutional review board statement

This study was approved by the Centro de investigación y Asistencia en Tecnología y Diseño del Estado de Jalisco A.C. (CIATEJ) Biosecurity Committee (First Ordinary session March 2021).

## Data Availability

The data supporting the findings of this study is available from the corresponding author upon reasonable request.
